# EIF4A3-mediated circPRKCI expression promotes triple-negative breast cancer progression by regulating WBP2 and PI3K/AKT signaling pathway

**DOI:** 10.1038/s41420-022-00892-y

**Published:** 2022-03-02

**Authors:** Xuehui Wang, Hongming Song, Lin Fang, Tianqi Wu

**Affiliations:** 1 Cancer Institute, Xuzhou Medical University, Xuzhou, Jiangsu China; 2 Department of Breast and Thyroid Surgery, Shanghai Tenth People’s Hospital, Tongji University School of Medicine, Shanghai, China; 3 Breast Disease Center, The Affiliated Hospital of Qingdao University, Qingdao, Shandong China

**Keywords:** Breast cancer, Oncogenes

## Abstract

Triple-negative breast cancer (TNBC) is known as a highly aggressive subtype of BC due to high rate of recurrence and metastasis, poor prognosis and lacking of effective targeted therapies. Circular RNAs (circRNAs) have been reported to participate in the progression of TNBC. In this study, we demonstrated that circPRKCI, derived from the *PRKCI* gene, was elevated in BC tissues and cell lines, especially in TNBC. The functional investigation showed that circPRKCI could significantly promote the proliferation and migration of TNBC in vivo and in vitro. In addition, circPRKCI regulated WBP2 and the phosphorylation of AKT via serving as miR-545-3p sponge. Of note, EIF4A3 could induce circPRKCI expression and nuclear export in TNBC cells. Taken together, EIF4A3-mediated circPRKCI could promote TNBC progression by regulating WBP2 and PI3K/AKT signaling pathway, providing a new avenue of therapy for TNBC.

## Introduction

Breast cancer (BC) has surpassed lung cancer as the most commonly diagnosed cancer all over the world in 2020 with highest incidence rate of 11.7% according to the International Agency for Research on Cancer (IARC) [1]. Triple-negative breast cancer (TNBC) is defined by lacking estrogen receptor (ER), progesterone receptor (PR), human epidermal growth factor receptor 2 (HER2) [2]. As a particularly aggressive subtype of BC, TNBC is known for its high rate of recurrence and metastasis, poor prognosis and lack of effective targeted therapies [3, 4]. Early diagnosis and intervention are crucial in minimizing recurrence and reducing treatment-associated morbidity of BC [5]. Therefore, it is still urgent to further seek the probable mechanism of the tumorigenesis and look for novel effective therapeutic targets of TNBC.

Circular RNAs (circRNAs) are a group of stable non-coding RNAs for their single-stranded, covalently loop structure without 5′ cap structure and 3′ poly-A tail [6]. Recently, circRNAs have been shown to participate in various stages of cancers, including tumorigenesis, progression, recurrence and metastasis [7, 8]. In addition, some specific circRNAs have been identified to play critical roles in the malignant progression of BC [9, 10]. For instance, cytoplasmic hsa_circ_0005273 was proved function as an oncogene in BC progression via regulating miR-200a-3p/YAP1 axis and inactivating hippo signaling pathway [11]. Hsa_circ_0068631, derived from gene *TFRC*, was unveiled to increase c-Myc mRNA stability by recruiting and binding to EIF4A3 [12]. Furthermore, due to their special covalently closed circular structures, circRNAs have great advantages in resisting degradation and have longer half-life time compared with the linear transcripts [13]. The superiority of stability provides circRNAs more advantages to act as potential biomarkers for BC diagnosis [14].

WW domain-binding protein 2 (WBP2), which was initially identified as a partner of Yes-associated protein (YAP), has been identified as an emerging oncogene in TNBC over the past decade [15, 16]. WBP2 was shown to promote TNBC migration and invasion via increasing BTRC mRNA stability and activating NF-κB [17]. Furthermore, WBP2 could initiate TNBC cells for responses to Wnt signaling via the JNK/Jun kinase pathway [18]. In addition, WBP2 was also correlative to PIK3/AKT pathway as it promoted TNBC proliferation by blocking YAP transcription and AKT phosphorylation [19]. Considering the vital role of WBP2 in various signaling pathways associated with TNBC progression, seeking an effective upstream regulator of WBP2 may be a potential therapy for TNBC.

In this study, we identified circPRKCI, also known as hsa_circ_0067934, was upregulated in both TNBC tissues and cell lines. CircPRKCI promoted the proliferation and migration of TNBC in vivo and in vitro via acting as a sponge of miR-545-3p, thereby upregulating WBP2 and promoting AKT phosphorylation. Furthermore, we found that EIF4A3 could induce circPRKCI expression and nuclear export. Our findings revealed a novel regulating mechanism of EIF4A3/circPRKCI/miR-545-3p/WBP2/AKT axis in TNBC, providing a new avenue of therapy for TNBC.

## Results

### Characteristics of circPRKCI in TNBC cells

**Fig. 1:**
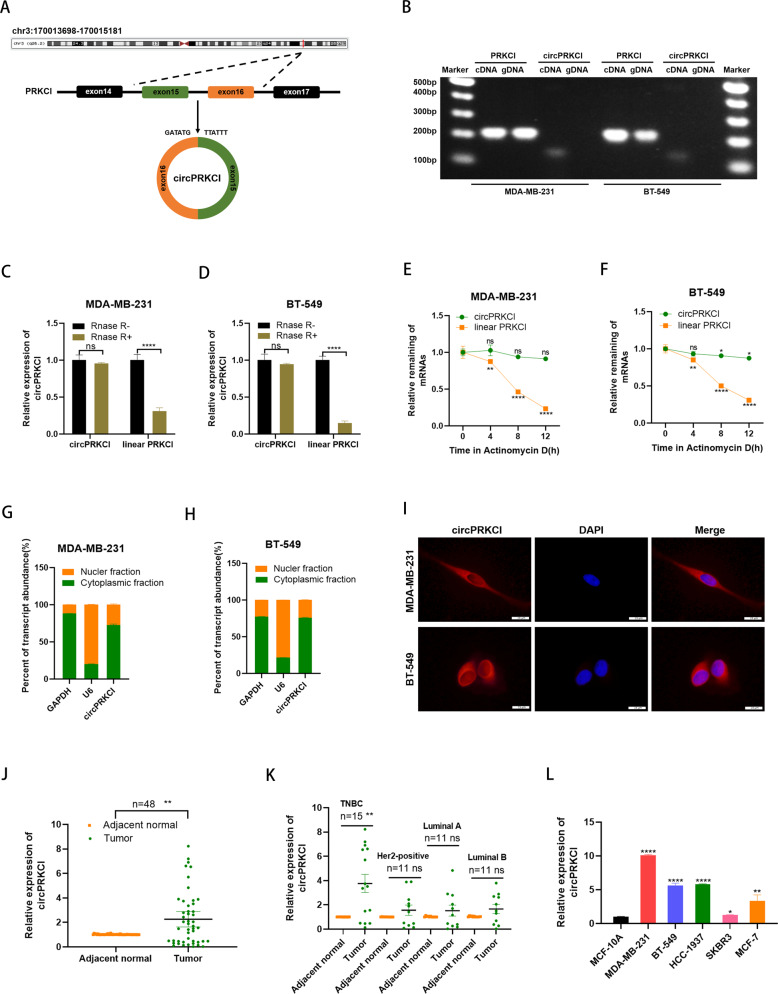
Characteristics of circPRKCI in TNBC cells. **A** CircPRKCI is formed by circularization of exon 15-16 of the gene PRKCI. **B** Existence of circPRKCI in TNBC cells was verified by agarose gel electrophoresis. **C**, **D** qRT-PCR analysis of circPRKCI and linear PRKCI in TNBC cells treated with RNase R. **E**, **F** After Actinomycin D treatment, the mRNA stability of circPRKCI and PRKCI in TNBC cells was determined by qRT-PCR. **G**, **H** Expression levels of cytoplasmic control transcripts (GAPDH), the nuclear control transcript (U6), and circPRKCI were determined by qRT-PCR in the cytoplasmic and nuclear fractions of TNBC cells. **I** RNA FISH for circPRKCI and nuclei was stained with DAPI. Red, circPRKCI; Blue, DAPI. **J** CircPRKCI was highly expressed in tumor tissues compared with adjacent normal tissues. **K** Expression of circPRKCI in TNBC cohort, Her2-positive cohort, luminal-A and luminal-B cohort, respectively. **L** Relative expression of circPRKCI in BC cell lines. *p < 0.05, **p < 0.01,****p < 0.0001.

In according to the UCSC Genome Browser Home (http://genome.ucsc.edu/), the 170bp-long circPRKCI was formed by back splicing of exon 15-16 of gene *PRKCI* (Fig. 1A). Then PCR assay indicated that divergent primers could produce circPRKCI with cDNA rather than with genomic DNA (gDNA), while convergent primers could produce the linear isoform of PRKCI with both cDNA and gDNA (Fig. 1B). RNase R treatment assay confirmed that circPRKCI could resist the digestion while the linear PRKCI was degraded by RNase R, indicating circPRKCI was a stable circular structure which was not easy to degrade (Fig. 1C, D). In addition, we treated MDA-MB-231 and BT-549 cells with Actinomycin D to inhibit transcription, and observed that circPRKCI had longer half-life time compared with linear PRKCI (Fig. 1E, F). Thereafter, the subcellular fractionation assay indicated that more than 70% circPRKCI located in cytoplasm of TNBC cells (Fig. 1G, H). Consistently, FISH assay in MDA-MB-231 and BT-549 cells also suggested that circPRKCI was mainly stained in cytoplasm of TNBC cells (Fig.1I). To explore the expression level of circPRKCI in BC tissues, we conducted qRT-PCR in 48 paired tissues from BC patients. The results showed that expression level of circPRKCI was significantly higher in BC tissues than in adjacent normal tissues (Fig. 1J). Moreover, 48 BC patients were divided into four cohorts based on their tumor subtype, including TNBC, Her2-positive, luminal-A and luminal-B cohorts. We further found that circPRKCI was particularly highly expressed in TNBC (Fig. 1K). Similarly, the expression level of circPRKCI was elevated in BC cell lines, especially in TNBC cell line MDA-MB-231, BT-549 and HCC-1937 (Fig. 1L). Additionally, as the highly aggressive subtype of BC, TNBC lacks effective targeted therapies. It is of great clinical significance to seek the probable mechanism of the tumorigenesis and look for novel effective therapeutic targets of TNBC. Taken together, we chose MDA-MB-231 and BT-549 cell line to further explore the biological function of circPRKCI in TNBC. Furthermore, the relationships between circPRKCI levels and clinical characteristics of 48 BC patients were also analyzed. We found the high expression level of circPRKCI was positive associated with TNM stage, tumor size and lymph node metastasis, but there were no significant associations with age, recurrence and metastasis, indicating that high circPRKCI expression was correlated with poor prognosis of patients with BC (Table 1).

**Table 1:** The relationship between the expression of circPRKCI and various clinicopathological variables in BC patients.

Patients Characteristics	Total	circPRKCI expression		*P* value
	(*n* = *48)*	High (*n* = *30)*	Low (*n* = *18)*	
Age				0.8229
<60	23	14	9	
≥60	25	16	9	
TNM stage				0.0352*
I and II	37	20	17	
III and IV	11	10	1	
Tumor size(cm)				<0.0001****
≤2	22	7	15	
>2	26	23	3	
Lymph node metastasis				0.0158*
Negative	29	14	15	
Positive	19	16	3	
Recurrence and metastasis				0.2631
No	46	28	18	
Yes	2	2	0	

*p < 0.05, ****p < 0.0001.

### CircPRKCI exerted oncogenic role in TNBC cells

**Fig. 2:**
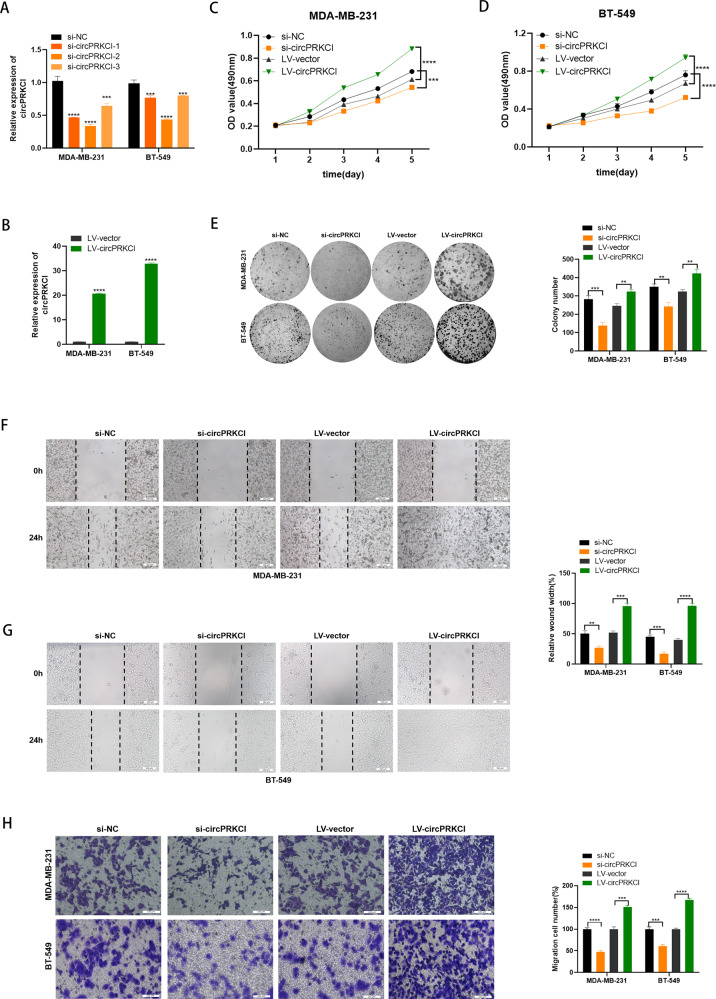
CircPRKCI exerted oncogenic role in TNBC cells. **A** Expression of circPRKCI was confirmed by qRT-PCR in BC cells transfected with si-NC or si-circPRKCI. **B** Expression of circPRKCI was confirmed by qRT-PCR in BC cells transfected with LV-vtctor or LV-circPRKCI. **C**, **D** Effect of si-circPRKCI and LV-circPRKCI on proliferation in TNBC cells by MTT assay. **E** Effect of si-circPRKCI and LV-circPRKCI on proliferation in TNBC cells by colony formation assay. **F**–**G** Effect of si-circPRKCI and LV-circPRKCI on migration in TNBC cells by wound healing assay. **H** Effect of si-circPRKCI and LV-circPRKCI on migration in TNBC cells by cell migration assay. **p < 0.01,***p < 0.001,****p < 0.0001.

To validate the biological function of circPRKCI in TNBC, we transfected TNBC cells with three specific siRNAs of circPRKCI (si-circPRKCI) and si-NC served as the negative control. qRT-PCR was used to verify the silencing efficacy of siRNAs. According to the results shown in Fig. 2A, si-circPRKCI-2 was used for further study. Also, we constructed circPRKCI plasmid (LV-circPRKCI) which could stably overexpress circPRKCI in TNBC cells compared to the control group (LV-vector) (Fig. 2B). The results of MTT assay showed that down-regulation of circPRKCI could inhibit the proliferation of TNBC cells, and the proliferation ability of TNBC cells was enhanced after increasing the expression level of circPRKCI (Fig. 2C, D). In addition, the colony formation assay confirmed the oncogenic role of circPRKCI in TNBC cells (Fig. 2E). Furthermore, the migration ability of MDA-MB-231 and BT-549 cells were promoted by circPRKCI confirmed by wound healing assay and transwell assay (Fig. 2F–H). Taken together, circPRKCI promoted proliferation and migration of TNBC cells.

### CircPRKCI served as a sponge for miR-545-3p

**Fig. 3:**
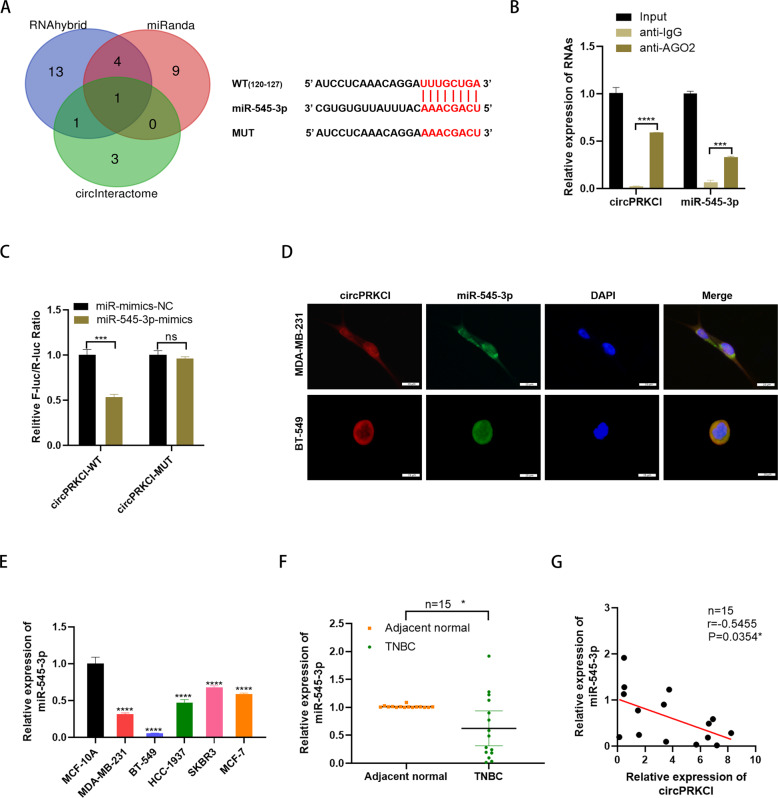
CircPRKCI served as a sponge for miR-545-3p. **A** Left, Venn diagram showing the potential target miRNAs of circPRKCI; Right, putative complementary sites within miR-545-3p and circPRKCI predicted by bioinformatics analysis. **B** RIP experiments were performed in HEK293T cells, and the co-precipitated RNA was subjected to qRT-PCR for circPRKCI and miR-545-3p. **C** Dual luciferase reporter assays demonstrated that miR-545-3p is a direct target of circPRKCI. **D** Detection of colocalization of circPRKCI and miR-545-3p in cytoplasm by RNA FISH assay (magnification, ×400). Green, miR-545-3p; Red, circPRKCI; Blue, DAPI. **E** miR-545-3p had low expression in BC cell lines. **F** miR-545-3p had low expression in TNBC tissues compared with adjacent normal tissues. **G** Correlations between the expression of circPRKCI and miR-545-3p were found with Pearson’s correlation analysis in TNBC tissue samples (*n* = 15). *p < 0.05,***p < 0.001,****p < 0.0001.

We assumed that circPRKCI might exert its tumor-promoting role in TNBC via acting as a miRNA sponge due to its cytoplasmic location. To identify the miRNAs that may have potential binding sites with circPRKCI, we examined three related circRNA databases, including RNAhybrid, miRNA and Circinteractome. As the Venn diagram showed, miR-545-3p was presumed have binding site with circPRKCI in all three databases, showing the highest possibility to directly bind to circPRKCI (Fig. 3A, left). Hence, we first conducted RIP assay with anti-AGO2, and the results suggested that circPRKCI was enriched in the miR-545-3p-AGO2 complex, suggesting that circPRKCI could bind to miR-545-3p in an AGO2 manner (Fig. 3B). Then, luciferase reporter assay was performed to further prove our hypothesis. According to the predicted binding sites of circPRKCI and miR-545-3p (Fig. 3A, right), we constructed WT and MUT plasmids containing complementary sequence. Compared with the negative control, miR-545-3p obviously decreased the relative activity (renilla/firefly) of the wild-type group, suggesting that miR-545-3p could bind from the 120^th^ to 127^th^ bases of circPRKCI (Fig. 3C). Additionally, FISH assay further showed circPRKCI and miR-545-3p co-localize in the cytoplasm of MDA-MB-231 and BT-549 (Fig. 3D). Thereafter, the expression level of miR-545-3p in BC was detected. Investigation by qRT-PCR demonstrated that miR-545-3p was down-regulated in BC tissues and cell lines (Fig. 3E, F). Importantly, miR-545-3p expression was negatively correlated with circPRKCI expression in TNBC tissues (Fig. 3G). Taken together, all results above showed that circPRKCI could directly target miR-545-3p.

### MiR-545-3p served as a tumor suppressor in TNBC cells

**Fig. 4:**
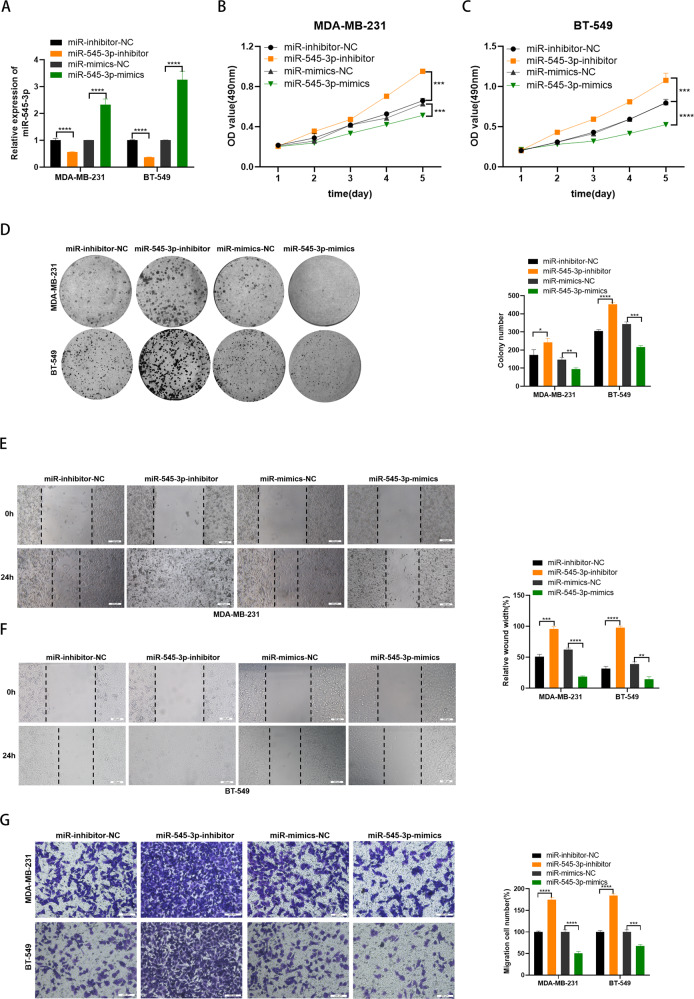
MiR-545-3p served as a tumor suppressor in TNBC cells. **A** Expression of circPRKCI was confirmed by qRT-PCR in TNBC cells transfected with miR-545-3p-inhibitor or miR-545-3p-mimics. **B**, **C** Effect of miR-545-3p on proliferation in TNBC cells by MTT assay. **D** Effect of miR-545-3p on proliferation in TNBC cells by colony formation assay. **E**, **F** Effect of miR-545-3p on migration in TNBC cells by wound healing assay. **G** Effect of miR-545-3p on migration in TNBC cells by cell migration assay. *p < 0.05, **p < 0.01,***p < 0.001,****p < 0.0001.

To clarify the biological function of miR-545-3p in TNBC, we transfected the inhibitor and mimics of miR-545-3p and the corresponding negative control into TNBC cells. The transfection efficiency was confirmed by qRT-PCR (Fig. 4A). Then functional experiments were performed to validate the potential function of miR-545-3p in TNBC cells. MTT proliferation assay and colony formation assay suggested that miR-545-3p could inhibit the proliferation of TNBC cells (Fig. 4B–D). Furthermore, wound healing assay and transwell assay supported that miR-545-3p impaired the migration of TNBC cells (Fig. 4E−G). These results suggested miR-545-3p serve as a tumor suppressor in TNBC cells.

### WBP2 was a direct target gene of miR-545-3p

**Fig. 5:**
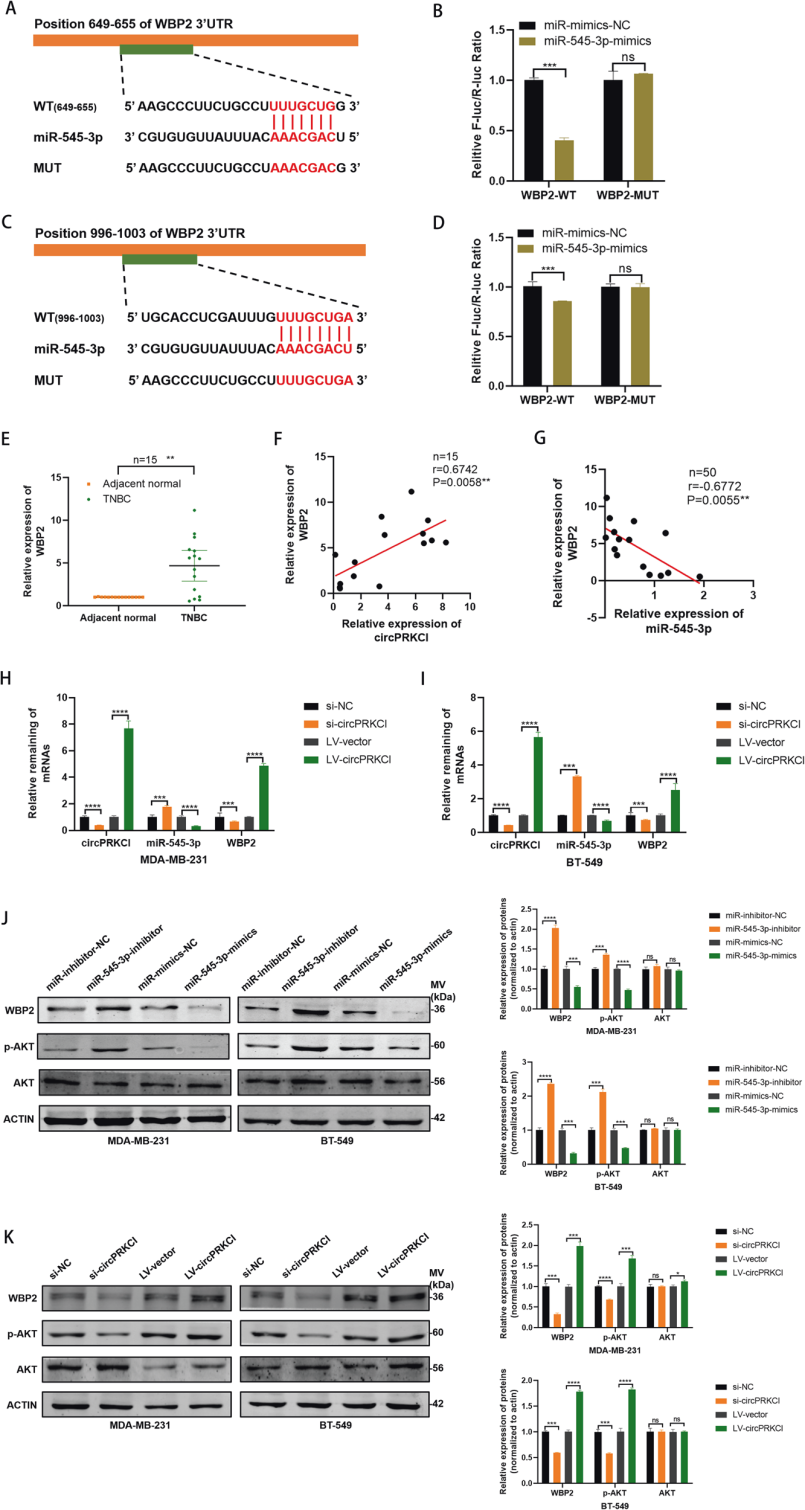
WBP2 was a direct target gene of miR-545-3p. **A**, **C** Putative complementary sites within miR-545-3p and WBP2 predicted by bioinformatics analysis (TargetScan). **B**, **D** Dual luciferase reporter assays demonstrated that WBP2 is a direct target of miR-545-3p. **E** WBP2 had high expression in TNBC tissues compared with adjacent normal tissues. **F** Positive correlations between the expression of circPRKCI and WBP2 were found with Pearson’s correlation analysis in TNBC tissue samples (*n* = 15). **G** Negative correlations between the expression of miR-545-3p and WBP2 were found with Pearson’s correlation analysis in TNBC tissue samples (*n* = 15). **H**–**I** The mRNA level of miR-545-3p and WBP2 were evaluated in TNBC cells transfected with si-circPRKCI and LV-circPRKCI. **J** The protein level of WBP2, AKT and p-AKT were evaluated in TNBC cells transfected with miR-545-3p-inhibitor or miR-545-3p-mimics. **K** The protein level of WBP2, AKT and p-AKT were evaluated in TNBC cells transfected with si-circPRKCI and LV-circPRKCI. *p < 0.05, **p < 0.01,***p < 0.001,****p < 0.0001.

Targetscan (http://www.targetscan.org/vert_72/) was used to identify the potential mRNAs that may have binding sites with miR-545-3p. WBP2 was considered a target gene of miR-545-3p with two potential complementary sites (Fig. 5A, C). Then luciferase reports plasmids were constructed according to the binding sites of miR-545-3p and 3′-UTR region of WBP2. The result of dual luciferase assay suggested that miR-545-3p could directly target WBP2 through two complementary sites (Fig. 5B, D). Thereafter, we detected the expression level of WBP2 and found that it is highly expressed in TNBC tissues (Fig. 5E). Importantly, the negative relevance between miR-545-3p and WBP2 and the positive relevance between circPRKCI and WBP2 were identified with Pearson’s correlation analysis (Fig. 5F, G). We then detected mRNA and protein level of WBP2 expression after inhibition or elevation of miR-545-3p. The results implied that both mRNA and protein level of WBP2 were contrary to the change trend of miR-545-3p, further indicating WBP2 was a downstream of miR-545-3p (Fig. 5H–J). Previous studies have suggested that the phosphorylation of AKT could be affected by WBP2, we further detected the general and phosphorylation of AKT [19]. As expected, the relative expression of phosphorylation of AKT has the same trend with WBP2 (Fig. 5J). Furthermore, the positive correlation between the expression level of WBP2 and circPRCKI was further confirmed in TNBC cells (Fig. 5H, I, K). Likewise, the expression level of phosphorylation of AKT suggested the consistent trend with WBP2 after knock down or overexpression of circPRCKI (Fig. 5K).

### CircPRKCI promoted TNBC progression via miR-545-3p/WBP2 axis

**Fig. 6:**
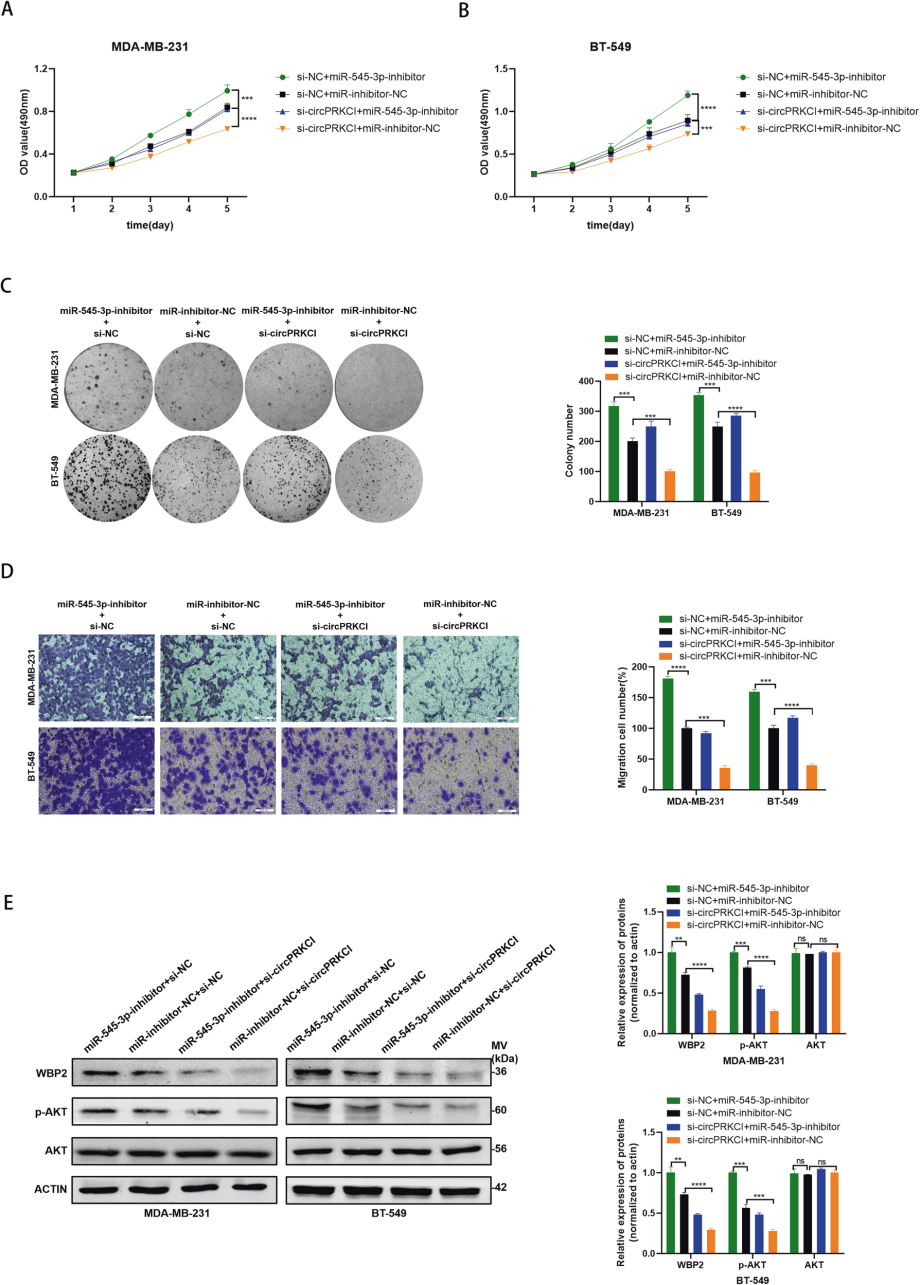
CircPRKCI promoted TNBC progression via miR-545-3p/WBP2 axis. **A**, **B** miR-545-3p-inhibitor rescued the promotive effects of circPRKCI in TNBC cells by MTT assay. **C** miR-545-3p-inhibitor rescued the promotive effects of circPRKCI in TNBC cells by colony formation assay. **D** miR-545-3p-inhibitor rescued the promotive effects of circPRKCI in TNBC cells by cell migration assay. **E** Western blotting showed that miR-545-3p-inhibitor can partly rescue the low expression of WBP2 and p-AKT caused by si-circPRKCI in TNBC cells. **p < 0.01,***p < 0.001,****p < 0.0001.

Results above have suggested the direct regulatory relationship of circPRKCI and miR-545-3p, as well as miR-545-3p and WBP2. Rescue assays were performed to further investigate whether circPRKCI exerts its oncogenic role via miR-545-3p/WBP2/AKT axis in TNBC. By co-transfecting si-circPRKCI and miR-545-3p inhibitor into TNBC cells, we found the co-transfection could partially eliminate the suppressive influence of si-circPRKCI on the proliferation and migration ability of TNBC cells (Fig. 6A–D). Consistently, the protein level of WBP2 and phosphorylation of AKT downregulated by si-circPRKCI were partially reversed by miR-545-3p inhibitor (Fig. 6E). Taken together, we verified that circPRKCI could regulate WBP2 and the phosphorylation of AKT via miR-545-3p to promote TNBC progression.

### EIF4A3 induced circPRKCI expression and nuclear export in TNBC cells

**Fig. 7:**
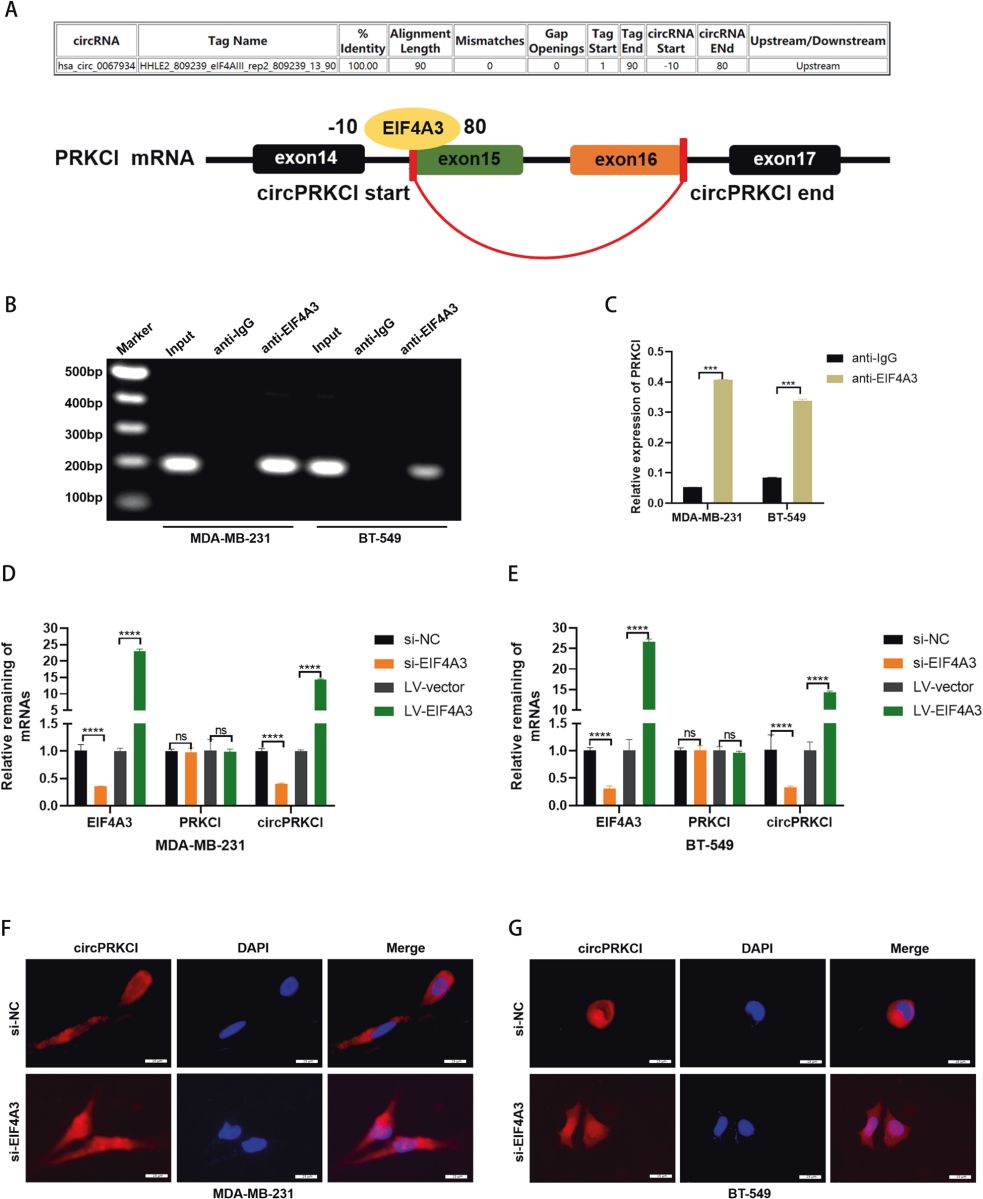
EIF4A3 induced circPRKCI expression and nuclear export in TNBC cells. **A** The binding sites of EIF4A3 in the flanking sequences of the PRKCI mRNA transcript were predicted. **B**, **C** RIP assay was applied to validate the binding of EIF4A3 to PRKCI mRNA. **D** and **E** The mRNA level of circPRKCI and PRKCI were evaluated in BC cells transfected with si-EIF4A3 and LV-EIF4A3. **F**, **G** RNA FISH for circPRKCI and nuclei was stained with DAPI in BC cells transfected with si-NC or si-EIF4A3. Red, circPRKCI; Blue, DAPI.

According to the prediction in Circular RNA Interactome (https://circinteractome.nia.nih.gov/), we found that EIF4A3 had a binding site with the upstream region of circPRKCI mRNA transcript (Fig. 7A). Previous studies have proved that EIF4A3, a core component of the exon junction complex, play an essential role in pre-mRNA splicing and could induce circular RNA formation via binding to the upstream region of some particular mRNAs 20. Therefore, we conduced RIP assay using anti-EIF4A3 antibody and confirmed the enrichment of PRKCI mRNA in EIF4A3 precipitates compared with the negative control (Fig. 7B, C). Later, we explored the effect of EIF4A3 on circPRKCI expression. We verified that overexpression of EIF4A3 induced circPRKCI rather than PRKCI mRNA expression, while knockdown of EIF4A3 reversed the inductive effect on circPRKCI expression in TNBC cells (Fig. 7D, E). In addition, we further explore whether EIF4A3 influences the subcellular localization of circPRKCI. After silencing the expression of EIF4A3 in TNBC cells, we found that most circPRKCI were stained in nucleus, suggesting that EIF4A3 promote the process of nuclear export of circPRKCI (Fig. 7F, G). To conclude, the data above suggested that EIF4A3 induced circPRKCI expression and nuclear export in TNBC cells.

### Overexpression of circPRKCI promoted TNBC tumor growth in vivo

**Fig. 8:**
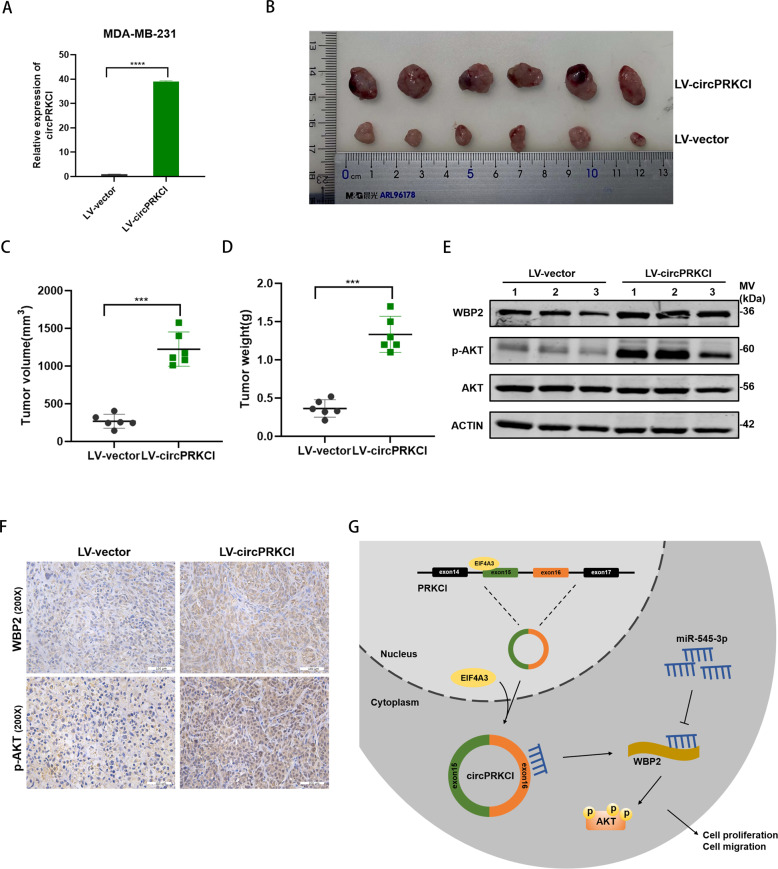
Overexpression of circPRKCI promoted BC tumor growth in vivo. **A** Overexpression of circPRKCI was confirmed by qRT-PCR in MDA-MB-231. **B** Representative images of xenograft tumors in nude mice (6 mice per group). **C** Average tumor volume of nude mice. **D** Average tumor weight of nude mice. **E** Extract protein from tumors and measuring the expression of WBP2, AKT and p-AKT by Western blotting. **F** Immunohistochemistry (IHC) staining of WBP2 and p-AKT in xenografts. **G** The mechanism diagram was generated to illustrate the mechanism of EIF4A3-circPRKCI-miR-545-3p-WBP2 axis in TNBC. ***p < 0.001,****p < 0.0001.

To further explore the biological function of circPRKCI in vivo, xenograft assay was carried out to observe the ability of MDA-MB-231 in promoting tumorigenesis with stably expression LV-circPRKCI. The efficiency of infection of LV-circPRKCI in MDA-MB-231 cells was ensued by qRT-PCR (Fig. 8A). Six weeks later, tumors dissected from sacrificed mice were photographed (Fig. 8B). Both the tumor volume and weight indicated that circPRKCI promoted the progression of TNBC in vivo (Fig. 8C, D). Thereafter, we analyzed the protein level of WBP2 and p-AKT from xenografts via western blotting and IHC. The relative protein expression of WBP2 and p-AKT were obviously upregulated in the circPRKCI overexpression group (Fig. 8E, F). To sum up, our research suggested that EIF4A3-mediated circPRKCI participate in the proliferation and migration of TNBC cells through circPRKCI/miR-545-3p/WBP2/AKT axis. The schematic diagram of the regulatory mechanism of circPRKCI was shown in Fig. 8G.

## Discussion

Despite great progress has been achieved in therapy for BC recent years, the clinical prognosis for TNBC unfortunately remain poor due to lacking of effective therapeutic targets. As a highly aggressive subtype of BC, TNBC is associated with younger age of onset, higher rate of recurrence and metastasis and lower overall survival rates [21, 22].

Recent years, numerous of circRNAs have been proved to participate in the tumorigenesis and progression of multiple cancers, including BC [23, 24]. Different from linear non-coding RNAs, circRNAs are a group of highly conservative and stable RNAs formed by back splicing [25]. Previous studies have revealed that the functions and mechanisms of circRNAs are commonly related to their subcellular localization [26]. Generally, most cytoplasmic circRNAs function as oncogenes and tumor suppressors through serving as miRNA sponges [27, 28], interacting with RNA-binding proteins [29, 30] and translation [31, 32], while circRNAs mainly located in nucleus could regulate transcription of their parental genes [33]. CircPRKCI, also known as circ_0067934, has been demonstrated to play critical roles in the tumorigenesis and progression of various cancers [34–37].

In this study, we found circPRKCI was highly expressed in TNBC tissues and cell lines. Subsequently, gain- and loss-of-function experiments in vitro suggested that circPRKCI promote the proliferation and migration of TNBC cells. Consistently, overexpression of circPRKCI promoted tumorigenesis of TNBC in vivo, further uncovering the oncogenic role of circPRKCI in TNBC. Considering that circPRKCI was mainly located in the cytoplasm of TNBC cells, we chose to explore its potential role in sponging miRNAs. Through bioinformatics analysis, we found miR-545-3p was a potential downstream miRNA of circPRKCI. Previous studies have suggested that miR-545-3p acts as a tumor suppressor in several cancers, including bladder cancer, gastric cancer and TNBC [38–40]. Thereafter, miR-545-3p was verified to directly bind to circPRKCI with RIP assay, dual-luciferase reporter assay and FISH-colocalization assay. Subsequently, the interaction between miR-545-3p and WBP2 3′-UTR was proved by dual-luciferase reporter assay, indicating that WBP2 was a direct target gene ofmiR-545-3p.

It is widely accepted that WBP2 is involved in multiple tumor-promoting signaling pathways and plays a dominant role in tumorigenesis [16, 41]. Additionally, WBP2 has been reported to regulate PI3K and AKT [16, 19]. Previous study demonstrated that PI3K/AKT signaling pathway plays a central role in TNBC oncogenic signaling, and the phosphorylation of AKT represents the activation of PI3K/AKT signaling pathway [42]. In our study, the relative expression of phosphorylation AKT has the same trend with WBP2 after knock down or overexpression of circPRCKI, which is in concert with previous study.

Recently, it has been reported that some RNA-binding proteins could influence circRNA production via binding to the flanking regions of circRNAs [43]. For example, EIF4A3 could promote circMMP9 expression via binding to MMP9 mRNA transcript [44]. Therefore, we further tried to explore the upstream of circPRKCI. Through bioinformatics prediction, we found EIF4A3 had a binding site on the upstream region of PRKCI mRNA transcript. As a core component of the exon junction complex (EJC), EIF4A3 plays a key role in precursor mRNA splicing, location, transport, translation, and degradation [45, 46]. Herein, we verified EIF4A3 induced circPRKCI expression by binding to PRKCI transcript. Furthermore, we found EIF4A3 also play a vital role in promoting nuclear export of circPRKCI.

## Conclusions

To sum up, we identified EIF4A3-mediated circPRKCI expression acts as a tumor-promoter in TNBC through regulating WBP2 and PI3K/AKT signaling pathway via serving as miR-545-3p sponge, providing a novel providing a new avenue of therapy for TNBC.

## Materials and methods

### Clinical samples

A total of 48 pairs of matched BC tissues and adjacent normal tissues were obtained from patients who underwent surgeries without any preoperative radiotherapy or chemotherapy in the Department of Breast and Thyroid, Shanghai Tenth People’s Hospital (Shanghai, China). All tissue samples were stored in liquid nitrogen immediately after resection until further use. All patients had given their consents and this study was approved by Institutional Ethics Committees of Shanghai Tenth People’s Hospital.

### Cell culture and transfection

All BC cell lines (MDA-MB-231 and BT-549) and normal breast epithelial cell lines (MCF-10A) were acquired from Chinese Academy of Sciences (Shanghai, China). All cell lines were authenticated and tested for mycoplasma contamination. The BC cell lines were cultured in Dulbecco’s Modified Eagle’s Medium (DMEM, Gibco, USA) with 10% Fetal Bovine Serum (FBS, Gibco, USA) and 1% penicillin-streptomycin (PS, Sigma, Germany). MCF-10A were cultured in Mammary Epithelial Basal Medium (MEBM) (Cambrex, USA). All cells were cultured in a 5% CO_2_ incubator at 37 °C. Hieff Trans^TM^ Liposomal Transfection Reagent (Yeasen, China) was used for transfection following the manufacturer’s instructions. Small interfering RNA targeting on circPRKCI (si-circPRKCI) and the negative control (si-NC) were purchased from IBSbio (Shanghai, China). Inhibitors, mimics and the negative control (miR-NC) for miR-545-3p were purchased from RiboBio (Guangzhou, China). Lentiviral plasmid for overexpressing circPRKCI (LV-circPRKCI) and the negative control (LV-vector) were designed by QiheBio (Shanghai, China).

### Confirming specificity for circPRKCI

Polymerase chain reaction (PCR) was performed with 2 × Hieff® Robust PCR Master Mix (YEASEN, China) and the PCR products amplified by circPRKCI primers were separated on 1% agarose gel. The gel was scanned by the Gel Doc XR + imager (Bio-Rad, USA).

### RNase R treatment

RNAs extracted from MDA-MB-231 and BT-549 cells were treated with Ribonuclease R (RNase R) at 37 °C for 30 min for enzyme inactivation, then RNAs were detected by qRT-PCR.

### Actinomycin D assay

MDA-MB-231 and BT-549 cells were treated with 2 μg/mL actinomycin D (Merck, Germany) to block transcription at 0 h, 4 h, 8 h and 12 h, respectively. Then remaining RNAs extracted from treated cells was assessed by RT‐qPCR.

### Subcellular fraction

Thermo Invitrogen™ PARIS™ Kit (Invitrogen, USA) was used for subcellular fraction according to the manufacturer’s instructions. U6 was used as nuclear control and GAPDH was used as cytoplasmic control.

### Fluorescent in situ hybridization (FISH)

Specific probe of circPRKCI for FISH was designed and synthesized by RiboBio (Guangzhou, China). Specific probe of miR-545-3p for FISH was designed and synthesized by IBSbio (Shanghai, China). Ribo™ Fluorescent In Situ Hybridization Kit (Ribo, China) was used to detect the localization of circPRKCI and miR-545-3p. The nucleus was stained by 4′,6-Diamidino-2-Phenylindole (DAPI). Fluorescence microscope (Leica, Germany) was applied in image acquisition.

### RNA extraction and quantitative real-time polymerase chain reaction (qRT-PCR)

Total RNA of tissues and cells were extracted by TRIzol reagent (Invitrogen, USA). The concentration and pureness of total RNA were determined by Nanodrop 2000 spectrophotometer (Thermo Fisher, USA). Hifair® III 1st Strand cDNA Synthesis SuperMix (Yeasen, China) was used to reverse RNA into cDNA. Hieff® qPCR SYBR® Green Master Mix (Yeasen, China) was used for qRT-PCR. 18 S, U6 and GAPDH were employed as the internal control for circRNA, miRNA and mRNA, respectively. Data were quantified by the 2^-ΔΔCt^ method. Primers used in this study were shown in Table S1.

### MTT assay

BC cells were seeded into 96-well plates at a density of 2000 cells per well with 200 μl medium. 20 μl MTT reagent (YEASEN, China) was added to each well at 0 h, 24 h, 48 h, 72 h and 96 h after seeding, respectively. After being incubated for 4–6 h, the supernatant was replaced by 150 μl DMSO (Sangon, China). Optical density (OD) at 490 nm was detected by a microplate spectrophotometer (BioTek, German).

### Colony formation assay

BC cells were seeded into 6-well plates at a density of 1000 cells per well. After being incubated for about 10 days until the colonies were visible. Cell colonies were subsequently washed with phosphate buffered saline (PBS), fixed with 95% ethanol and stained with 0.1% crystalline violet. The representative photographs were taken and the number of colonies were counted.

### Wound healing assay

BC cells were seeded into 6-well plates. When the cells reached about 95% confluent, A scratch was produced on the surface of the cell monolayers with a 200 μl pipette tip. Then the cells were cultured with DMEM medium with 2%FBS. The healing of the wound at the same position was observed and assessed with a microscope at 0 h and 24 h.

### Transwell assay

BC cells were added into the upper chamber with 200 μl serum-free medium and medium with 10% FBS was added into the lower chamber. After culturing for 16 h for MDA-MB-231 cell line and 18 h for BT-549 cell line, cells migrated to the opposite side of the filter were fixed, stained, photographed (Leica Microsystems, Germany) and counted.

### Dual-luciferase reporter assay

Wild type (WT) and mutant (MUT) reporter plasmids of both circPRKCI and WBP2 3′-UTR were constructed according to the predicted sequence of the binding sites with miR-545-3p. MiR-545-3p mimics or miR-545-3p NC was co-transfected with WT or MUT reporter plasmids into HEK-293T cells. After been incubated for about 48 h, total protein from cell lysates was collected and centrifuged and dual-luciferase reporter assay kit (YEASEN, China) was used to detect the luciferase activities. Then the ratio of firefly to renilla luciferase was calculated.

### RNA Immunoprecipitation Assay (RIP)

BersinBio^TM^ RNA Immunoprecipitation Kit (BersinBio, China) was used for conducting RIP assay according to the manufacturer’s instructions. Anti-Ago2 (Abclonal, China), anti-EIF4A3 (Proteintech, China) or anti-IgG (Abclonal, China) were utilized for immunoprecipitation. The enriched RNAs were further analyzed by qRT-PCR.

### Protein extraction and western blotting analysis

Total proteins were extracted by RIPA lysis buffer (Beyotime, China) together with PMSF (Beyotime, China). The concentration of total protein was determined by BCA protein assay kit (Beyotime, China). After being separated by 10% sodium dodecyl sulfate-polyacrylamide (SDS-PAGE) gels, proteins lysates were transferred to nitrocellulose membranes (Beyotime, China). The membranes were blocked with 5% non-fatty milk for 1 h at room temperature and then immunoblotted at 4 °C overnight with primary antibodies: anti-WBP2 (1:1000, Proteintech, USA), anti-AKT (1:1000, Proteintech, USA), anti-p-AKT (1:1000, Abcam, USA) and anti-β-actin (1:10,000, Abclonal, China). After incubated with diluted secondary antibodies for 1 h at room temperature, the bands were scanned and analyzed by Odyssey Infrared scanning system (LI-COR Biosciences, USA). Original western blots were shown in Fig. S1.

### Xenografts experiment

4-week-old female BALB/c nude mice were ordered from SLAC (Shanghai, China) and divided into two groups randomly (*n* = 6, each group). A total of 1 × 10^6^ transfected MDA-MB-231 cells with stably expressed circPRKCI or LV-vector were injected into the second mammary fat pad of mice in two groups, respectively. After 6 weeks, all mice were sacrificed by cervical dislocation and collected tumor volume was measured and calculated as follows: Volume (mm^3^) = width^2^ × length/2. The animal experiments complied with the rule of the ethics committee of Shanghai Tenth People’s Hospital of Tongji University.

### Immunohistochemistry (IHC)

Tumor tissue collected from the BALB/c nude mice was fixed in 4% paraformaldehyde, dehydrated by ethanol solution, embedded in paraffin and sectioned into 4 μm slides. Then the slides were incubated with anti-WBP2 (Proteintech, USA) and anti-p-AKT (Abcam, USA). Images were photographed by Leica Microsystems (Germany).

### Statistical analysis

Data obtained from at least three independent experiments were analyzed by GraphPad Prism (v8.3.0, USA). Data were presented means ± standard deviation (SD) and considered as significant when *P*-values < 0.05. Relationships between the expression of circPRKCI and various clinicopathological variables were analyzed by chi-square test and Fisher’s exact test. Comparisons between paired specimens were analyzed by Wilcoxon matched-pairs signed-rank test. Unpaired samples were analyzed by unpaired Student’s *t*-test.

## Supplementary information

Table S1

Figure S1

Author contribution statement

## Data Availability

The circRNAs sequence data were obtained from circbase (http://circrna.org/).
