# Supplementary material for: EIF4A3-mediated circPRKCI expression promotes triple-negative breast cancer progression by regulating WBP2 and PI3K/AKT signaling pathway
Source: Cell Death Discov. 2022 Mar 2;8:92. doi: 10.1038/s41420-022-00892-y (PMC8891274; doi:10.1038/s41420-022-00892-y)
Supplement: Supplementary file 1 — Table S1 [file 41420_2022_892_MOESM1_ESM.docx]

**Table S1 Primers and siRNAs used in this study**

| **Gene** | **Sequences** |
| --- | --- |
| 18S-F  18S-R | 5'-TAGAGGGACAAGTGGCGTTC-3'  5'-CGCTGAGCCAGTCAGTGT-3' |
| circPRKCI-F | 5′-TAGCAGTTCCCCAATCCTTG-3′ |
| circPRKCI-R  GAPDH-F  GAPDH-R | 5′-GATCCATGGGCATCACTGGT-3′  5'-CAGGAGGCATTGCTGATGAT-3'  5'-GAAGGCTGGGGCTCATTT-3' |
| PRKCI-F | 5′-GCCACACTTTCCAAGCCAAG-3′ |
| PRKCI-R | 5′-GATCCATGGGCATCACTGGT-3’ |
| WBP2-F | 5’-GCGGAGTGATCGTCAATAACA -3' |
| WBP2-R | 5'- GACCCGGTAAGGGGTAAGGT-3' |
| miR-545-3p-F | 5’-TCAGTAAATGTTTATTAGATGA-3’ |
| miR-545-3p-R | 5’-GTGCAGGGTCCGAGGTATTC-3’ |
| U6-F | 5'-CAAATTCGTGAAGCGTTCCATAT-3' |
| U6-R | 5'-GCTTCACGAATTTGCGTGTCATCCTTGC-3' |
| si-circPRKCI-1-Sense | 5’-GAUUGGGAUAUGUUAUUUUGGTT-3’ |
| si-circPRKCI-1-Anti-sense | 5’-CCAAAAUAACAUAUCCCAAUCTT -3’ |
| si-circPRKCI-2-Sense | 5′-GGGAUAUGUUAUUUUGGAAAATT-3′ |
| si-circPRKCI-2-Anti-sense | 5′-UUUUCCAAAAUAACAUAUCCCTT-3’ |
| si-circPRKCI-3-Sense | 5’-UGUUGAUUGGGAUAUGUUAUUTT-3' |
| si-circPRKCI-3-Anti-sense | 5'-AAUAACAUAUCCCAAUCAACATT-3' |
| si-NC-Sense | 5’-UUCUCCGAACGUGUCACGUTT-3’ |
| si-NC-Anti-sense  si-EIF4A3-Sense  si-EIF4A3-Anti-sense | 5’-ACGUGACACGUUCGGAGAATT-3’  5'-CCUCCAGUGUUUGGAUAUUTT-3'  5'- AAUAUCCAAACACUGGAGGTT-3' |
