# Supplementary figures and images for: EIF4A3-mediated circPRKCI expression promotes triple-negative breast cancer progression by regulating WBP2 and PI3K/AKT signaling pathway
Source: Cell Death Discov. 2022 Mar 2;8:92. doi: 10.1038/s41420-022-00892-y (PMC8891274; doi:10.1038/s41420-022-00892-y)

Fig.5J

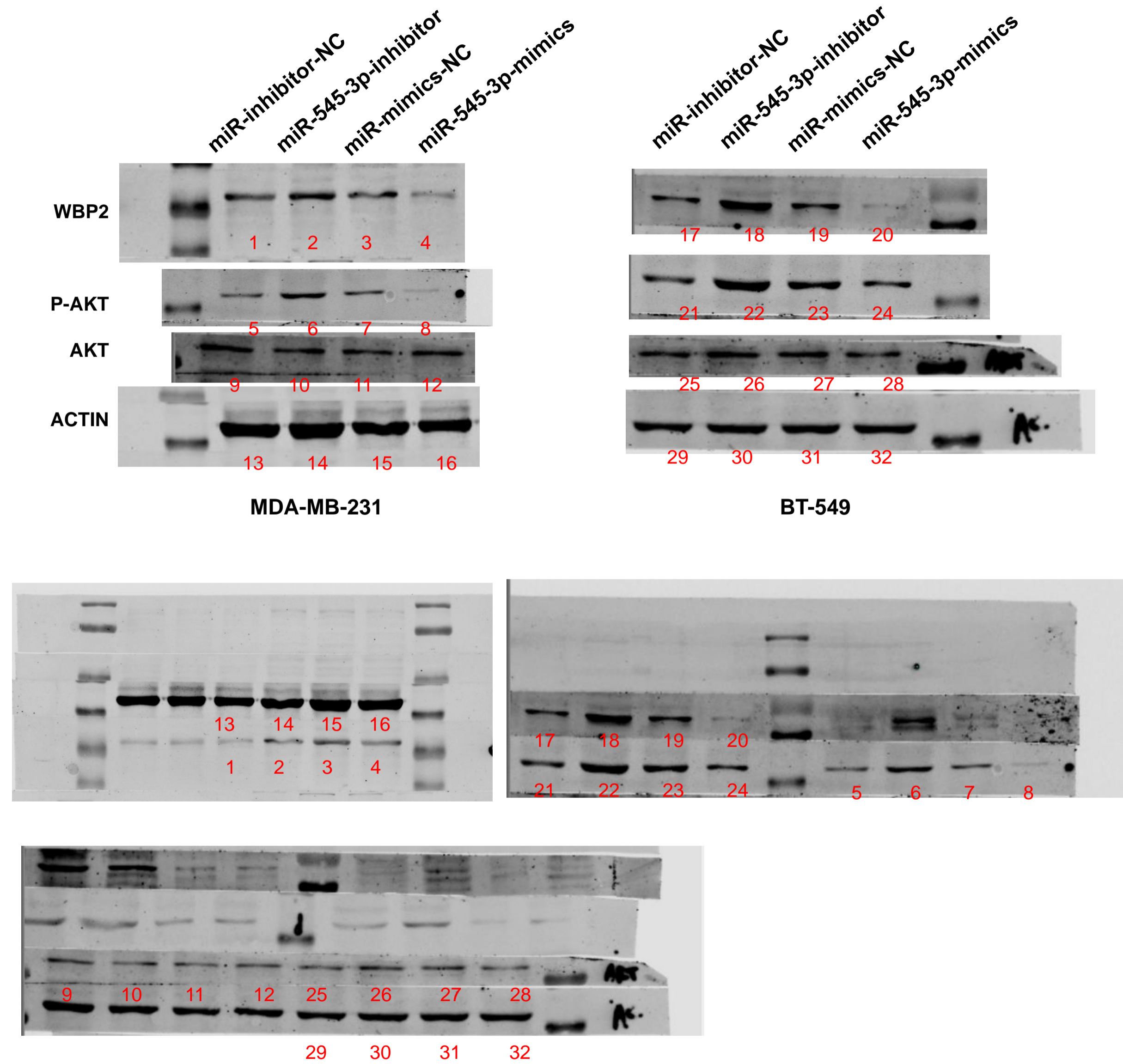

Fig.5K

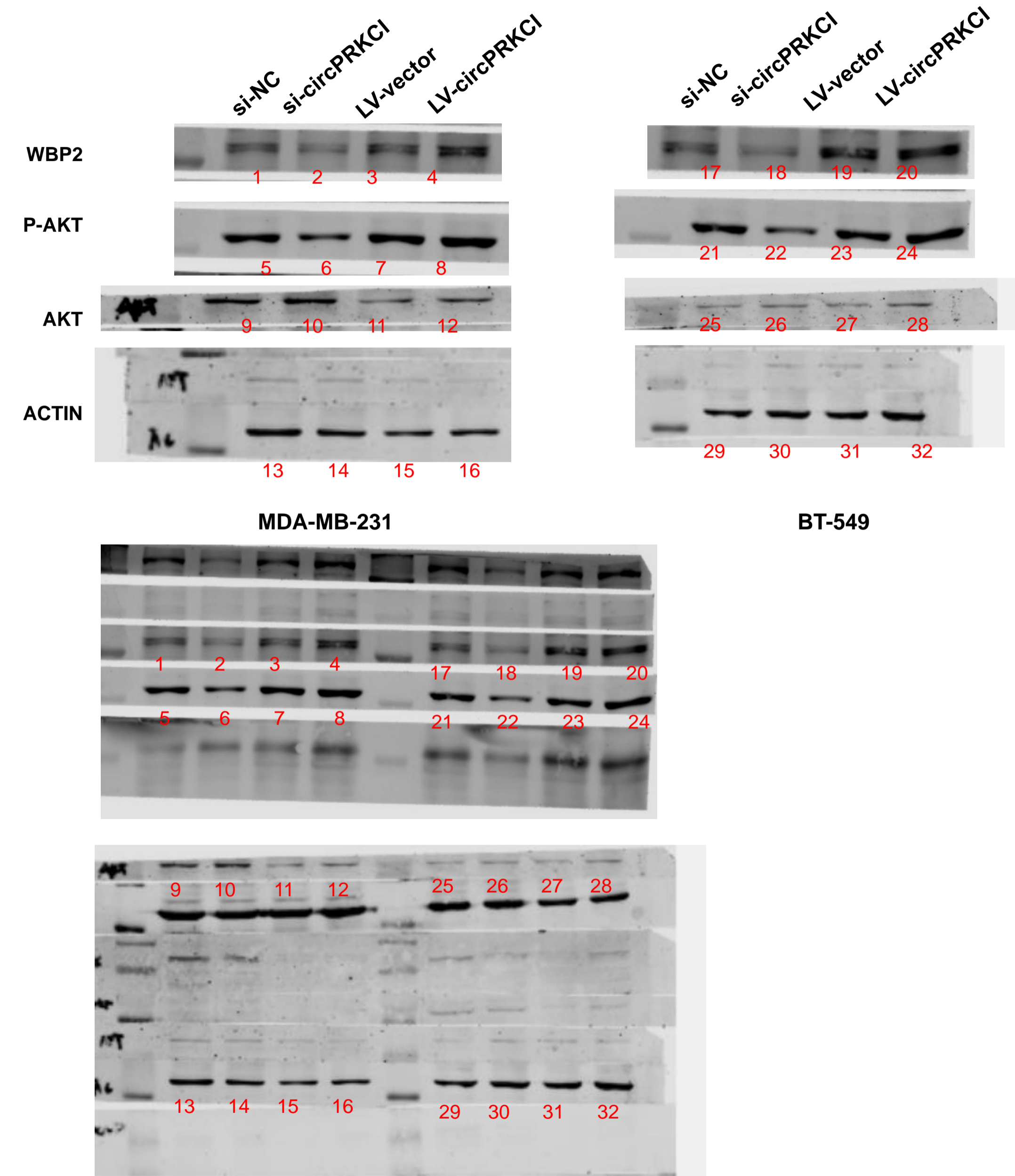

Fig.6E

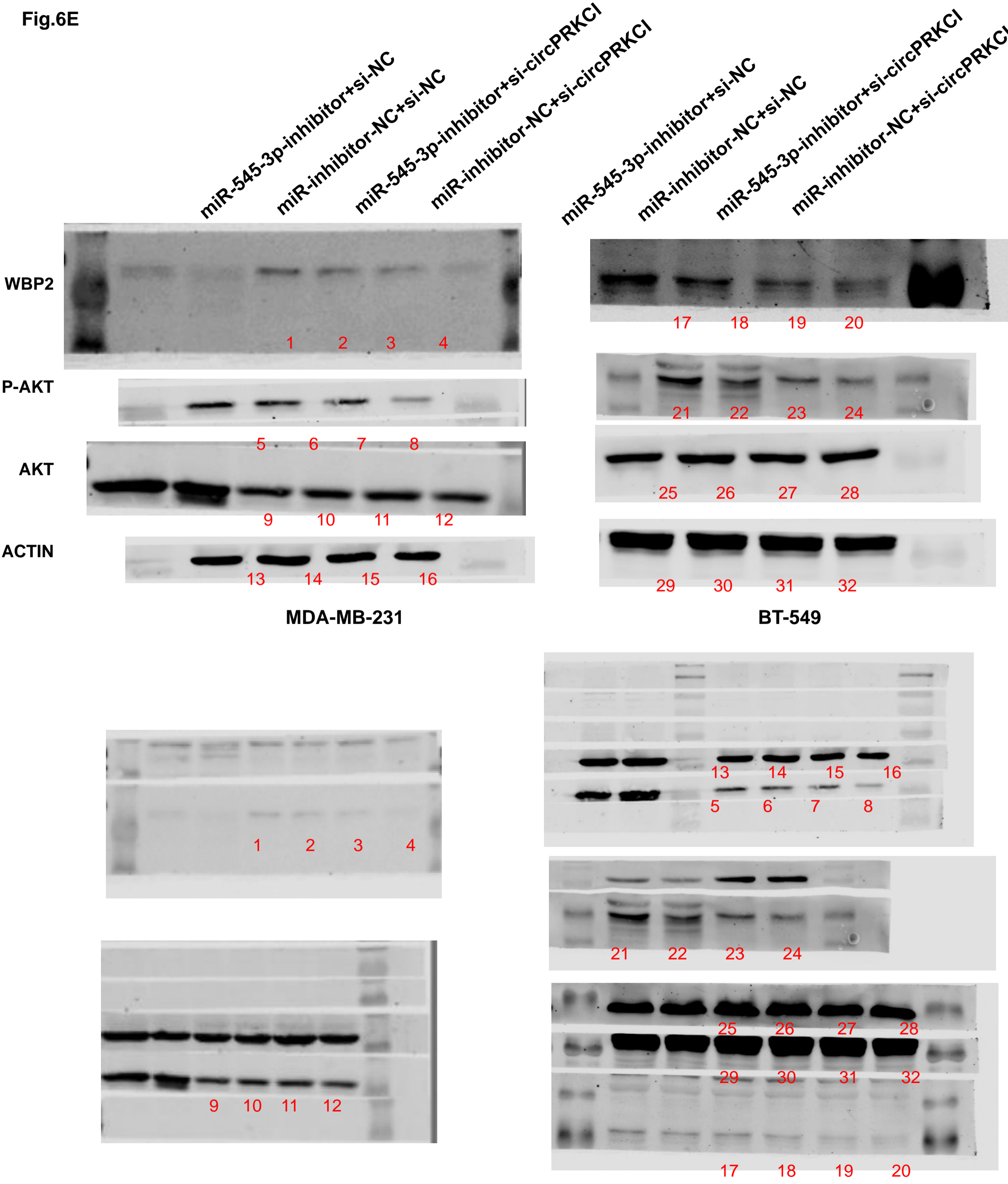

Fig.8E

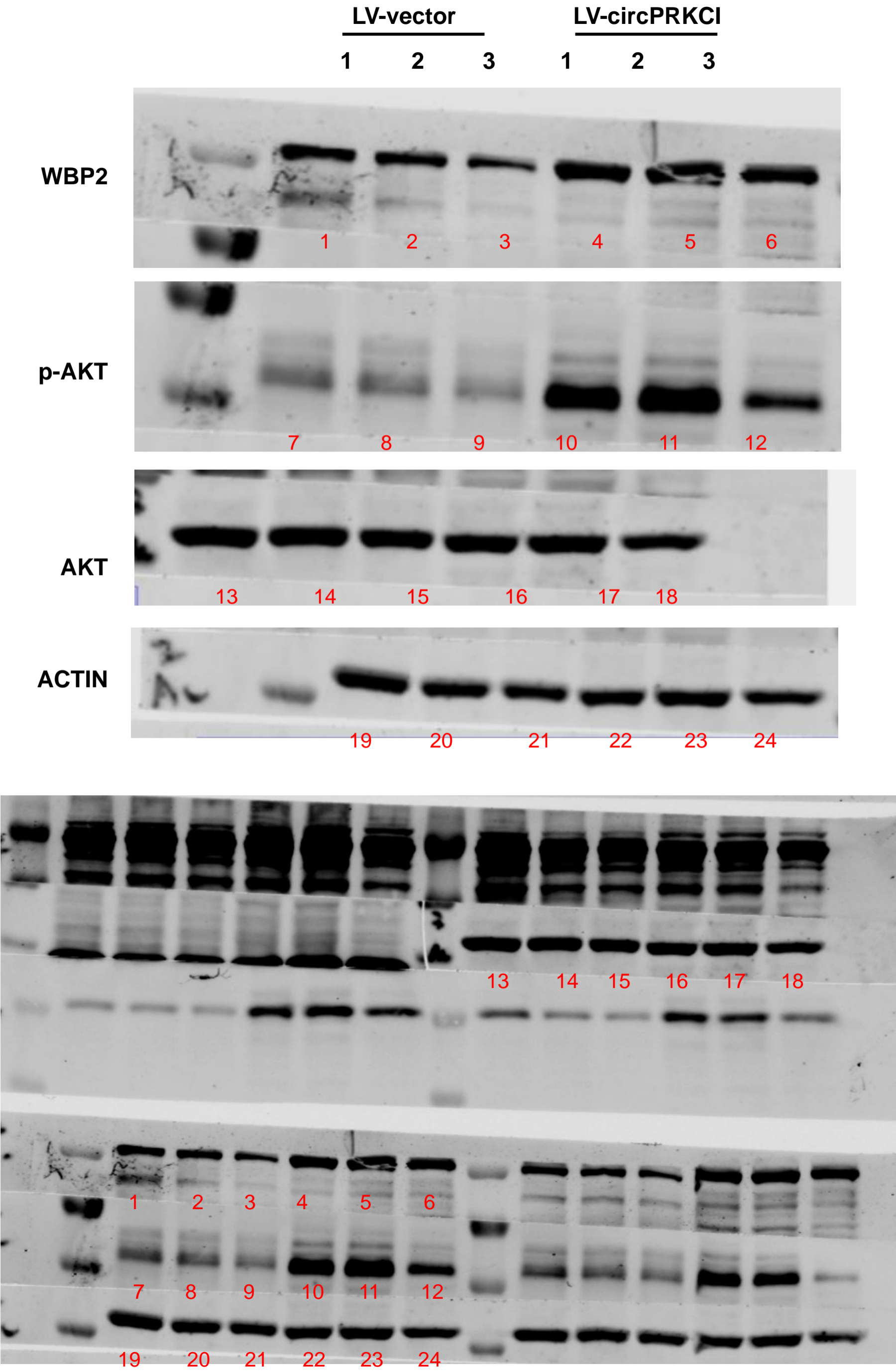

Supplement: Supplementary file 2 — Figure S1 [file 41420_2022_892_MOESM2_ESM.pdf]
