# Supplementary material for: EIF4A3-mediated circPRKCI expression promotes triple-negative breast cancer progression by regulating WBP2 and PI3K/AKT signaling pathway
Source: Cell Death Discov. 2022 Mar 2;8:92. doi: 10.1038/s41420-022-00892-y (PMC8891274; doi:10.1038/s41420-022-00892-y)
Supplement: Supplementary file 3 — Author contribution statement [file 41420_2022_892_MOESM3_ESM.pdf]

AUTHOR CONTRIBUTIONS

XW, LF and TW designed the research. XW performed the research and analyzed results. XW wrote the paper. HS edited the manuscript and provided critical comments. All authors read and approved the final manuscript.
